# Premotor cortex is critical for goal-directed actions

**DOI:** 10.3389/fncom.2013.00110

**Published:** 2013-08-12

**Authors:** Christina M. Gremel, Rui M. Costa

**Affiliations:** ^1^Laboratory for Integrative Neuroscience, National Institute on Alcohol Abuse and Alcoholism, National Institutes of HealthBethesda, MD, USA; ^2^Champalimaud Neuroscience Programme, Champalimaud Institute for the UnknownLisbon, Portugal

**Keywords:** premotor cortex, goal-directed actions, habitual actions, value-based decision making, action selection

## Abstract

Shifting between motor plans is often necessary for adaptive behavior. When faced with changing consequences of one’s actions, it is often imperative to switch from automatic actions to deliberative and controlled actions. The pre-supplementary motor area (pre-SMA) in primates, akin to the premotor cortex (M2) in mice, has been implicated in motor learning and planning, and action switching. We hypothesized that M2 would be differentially involved in goal-directed actions, which are controlled by their consequences vs. habits, which are more dependent on their past reinforcement history and less on their consequences. To investigate this, we performed M2 lesions in mice and then concurrently trained them to press the same lever for the same food reward using two different schedules of reinforcement that differentially bias towards the use of goal-directed versus habitual action strategies. We then probed whether actions were dependent on their expected consequence through outcome revaluation testing. We uncovered that M2 lesions did not affect the acquisition of lever-pressing. However, in mice with M2 lesions, lever-pressing was insensitive to changes in expected outcome value following goal-directed training. However, habitual actions were intact. We confirmed a role for M2 in goal-directed but not habitual actions in separate groups of mice trained on the individual schedules biasing towards goal-directed versus habitual actions. These data indicate that M2 is critical for actions to be updated based on their consequences, and suggest that habitual action strategies may not require processing by M2 and the updating of motor plans.

Adaptive behavior requires the ability to change motor plans depending on the consequence of actions. It has been previously shown that distinct instrumental processes—goal-directed versus habitual—can be used during action selection (Adams and Dickinson, 1981). In goal-directed behavior actions are selected based on the causal relation between their performance and expected consequences (outcomes); i.e., changing the value of the expected outcome of an action would change the probability of selecting that action. In contrast, habitual or automatic actions are thought to depend more on the history of reinforcement of that action and less on the expected consequences at the moment of selection (Dickinson, 1985; Colwill and Rescorla, 1986). The performance of well-learned actions in an automatic or habitual manner may be very efficient for daily functioning. However, changing circumstances can alter the expected consequence of actions, and in those situations it may be advantageous to be able to control which actions to take in a goal-directed manner.

The distinction between goal-directed and habitual actions can be seen in the different control over actions by expected outcome value following random ratio (RR) and random interval (RI) schedule training, respectively (Adams and Dickinson, 1981; Adams, 1982; Dickinson et al., 1983; Colwill and Rescorla, 1985). Historically, RR schedules bias towards use of goal-directed action strategies with a strong correlation between action rate and reward rate. In contrast, the uncertainty in the action-reward contiguity found in RI schedules biases towards the use of habitual action strategies (Derusso, 2010). Recently, we have shown that mice concurrently trained on RR and RI schedules will readily shift between goal-directed and habitual action strategies (Gremel and Costa, 2013).

Previous work has suggested that the pre-supplementary motor area (pre-SMA) in primates is involved not only in the learning of new actions, but also in the updating of motor plans based on the consequences of the actions, as evidenced by involvement in response inhibition, action switching and action timing (Rushworth et al., 2002; Hoshi and Tanji, 2004; Isoda and Hikosaka, 2007; Obeso et al., 2013). In this study, we investigated whether goal-directed action strategies, which require control based on changes in expected outcome value, depend on premotor cortex function. We evaluated the effects of lesions of the premotor cortex (M2) in mice—which is thought to be roughly equivalent to primate pre-SMA (Yin, 2009; Sul et al., 2011)—on the content of learning in an appetitive single-lever pressing task. We concurrently trained mice to make a very similar lever-press (same lever, same location) for the same food reward using a goal-directed versus habitual action strategy. We found that M2 lesions only disrupted actions controlled by the expected outcome value. We confirmed this in separate groups of M2 lesion mice trained only to make goal-directed or only habitual actions. These findings show that while goal-directed actions depend upon M2, habitual action strategies are executed independently of processing by M2 and suggest the automatic actions do not require updating of motor plans.

## Materials and Methods

### Mice

Male C57Bl/6J mice (*n* = 45) were purchased from The Jackson Laboratory (Harbor, ME), and at least eight weeks of age at the start of experiments. All procedures were approved by the NIAAA ACUC and done in accordance with NIH guidelines.

### Surgery and histology

Mice were anesthetized with isofluorane (1–2%) to stereotaxically (Kopf, CA, USA) target anterior M2 (from Bregma (mm); anteroposterior +1.34, mediolateral ±0.75, and dorsoventral (relative to skull) −1.25). To induce bilateral excitotoxic lesions to M2, ibotenic acid 0.3 μl (10 mg/ml in saline) was infused via Hamilton syringe (0.05 μl/min/side) or injector connected to a pump (Razel, Scientific) (0.1μl/min/side). Ibotenic acid was used because it lesions local neurons while sparing fibers of passage. For Sham mice, the Hamilton syringe or injector was lowered to the target site but no infusion was given. Mice were allowed to recover for at least 10 days before the start of behavioral procedures. Post-experimental procedures mice were perfused and brains post-fixed with 4% w/v paraformaldehyde, with lesion placement identified through Nissl staining of 50 μm brain slices.

### Behavioral Procedures

All behavioral training and testing generally took place as previously described (Hilário, 2007). In brief, mice were placed in operant chambers housed in sound attenuating boxes (Med-Associates, St. Albans, VT) and trained to press a single lever (left or right of a central food magazine) that was present the entire duration of the session. Mice lever-pressed for an outcome of either regular “chow” pellets (20 mg pellet, Bio-Serve formula F05684) or sucrose solution (20–30 μl of 20% solution) that were delivered into the food magazine. The other outcome was provided later in their home-cage and used as a control for general satiation in the revaluation test. Before training commenced, mice were food restricted to 90% of their baseline weight at which they were maintained for the duration of experimental procedures. Water was available at all times in the home cage.

### Within-subject design

**Figure 1:**
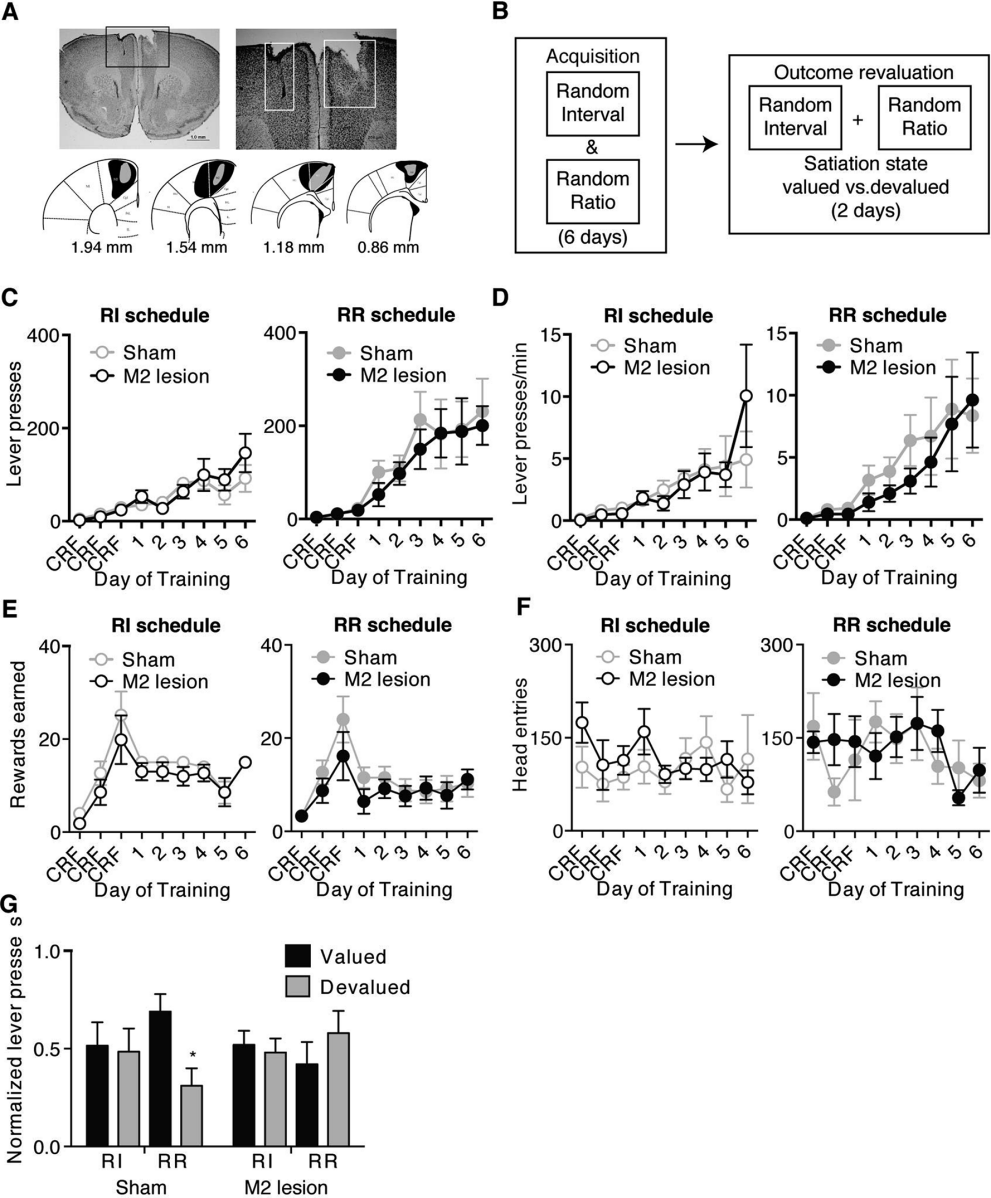
**M2 lesions disrupt the ability to shift between automatic and goal-directed actions in the same animal.** Representative picture of an M2 lesion **(A)**, with the area of lesion outlined in the box in the left panel enlarged in the right panel. Bottom panels are illustrated examples showing approximately the largest (black) and smallest (grey) extent of the lesions observed. **(B)** Schematic of experimental design. During acquisition, mice were concurrently trained under random interval (RI) and random ratio (RR) reinforcement schedules to press a similar lever for the same outcome. A separate outcome was provided daily in the home cage. Mice then underwent outcome revaluation testing comprising Valued (pre-fed home-cage outcome) and Devalued (pre-fed operant outcome) days. **(C–F)** Lever-pressing behavior during acquisition of concurrent RI (left panel) and RR (right panel) schedules, showing the effects of M2 lesions on the number of lever presses made **(C)**, the rate of lever pressing **(D)**, the number of rewards earned **(E)**, or head entries **(F)** during RI and RR schedule training. Mice then underwent subsequent outcome revaluation testing. **(G)** Shown is normalized lever pressing [Lever presses for each Revaluation state (Valued or Devalued state)/total Lever presses (Valued + Devalued states)] during outcome revaluation testing for Sham and M2 lesion mice. Non-reinforced lever pressing in previously RI and RR training contexts was examined on both Valued (black bars) and Devalued (grey bars) days. Error bars = ± SEM. * = Bonferroni corrected *p* < 0.05.

Mice were trained to shift between goal-directed and habitual actions strategies using a recently developed within-subject design (Figure 1B) (Gremel and Costa, 2013). We used different schedules of reinforcement to bias use of different action strategies; RR schedules were used to bias acquisition of goal-directed actions, while RI schedules were used to bias development of habitual actions. In RR (X) schedules, reinforcement follows after an average number (X) of actions have been made. Under RI (Y) schedules, the first action after an average time period (Y) has passed is reinforced. A probability distribution of *p* = 0.10 was used for all schedules. For example, in an RI60 schedule, on average one reinforcer is delivered upon the first press after 60 sec since the last reinforcer. For an RR20 schedule, on average one reinforcer is delivered ever 20 lever presses. Each day mice were trained in two separate operant chambers distinguished by contextual cues (black and white striped walls vs. clear plexiglass). For each mouse, the order of schedule exposure, lever position and the outcome obtained upon lever press were kept constant across contexts. However, mice were counterbalanced for context, schedule order, lever position, and outcome earned. Each training session commenced with illumination of the house light and lever extension, and ended following schedule completion or after 60 min with the lever retracting and the house-light turning off.

On the first day, mice were trained to approach the food magazine (no lever present) to retrieve a food outcome in each context on a random time (RT) schedule, with an outcome delivered on average every 60 sec for a total of 15 min. Next, in the absence of any predictive stimuli (e.g., cue light) mice were trained on continuous reinforcement schedules (CRF) in each context, where every lever-press made was reinforced with the same outcome, with the possible number of earned outcomes increasing across training days (5, 15, and 30 outcomes). After acquiring lever-press behavior, mice were trained on RI30 and RR10 schedules of reinforcement for two days, followed by four days of RI60 and RR20 schedule training. Schedules were differentiated by context, with the possibility of earning 15 outcomes in each context. The session ended after delivery of the 15th outcome or after 60 min had elapsed, with the lever retracting and the house light extinguishing.

Acquisition training was followed by an outcome revaluation test, in which sensory-specific satiation was used to probe the degree to which an action in each training context was sensitive to changes in value (Adams and Dickinson, 1981; Hilário, 2007). Testing was conducted across two days, the Valued day and the Devalued day. In brief, on the Valued day, mice had 1 hr *ad libitum* access to the home-cage outcome. On the Devalued day, mice were given 1 hr *ad libitum* access to the outcome previously earned by lever-press. Following pre-feeding on both Valued and Devalued days, mice were given brief (5 min) non-reinforced probe tests in RI and RR training contexts, and lever-press behavior was examined. Order of context exposure during testing was the same as training exposure, with order of revaluation day (Valued vs. Devalued) counterbalanced across mice.

### Individual-schedule training

Acquisition proceeded as described above in the within-subject design, except that each group of mice was trained in a single context on either a RR or RI schedule of reinforcement. On the first day, mice were trained to approach the food magazine (no lever present) on a RT schedule, with a reinforcer delivered on average every 60 sec for a total of 15 min. Mice were then trained on a CRF schedule, with the potential earned rewards increasing across three days (5, 15, and 30 potential rewards). After acquiring lever-press behavior, mice were trained either on a RI (two days of RI30 followed by four days of RI60) or a RR (two days of RR10, followed by four days of RR20) schedule. To equate to the total number of possible outcomes earned during within-subject experiments, mice had the opportunity to earn 30 outcomes within a 60 min session, in which after 60 min had passed the lever-retracted and the house light is extinguished.

### Data analysis

Pre-planned repeated-measures ANOVAs were used to examine effects of Lesion group and Training day on lever-press related behaviors during acquisition under RI and RR schedules of reinforcement. Lever pressing during outcome revaluation testing was normalized (lever-presses for each revaluation state normalized to total lever-presses during Valued and Devalued revaluation states), and preplanned 2-way ANOVA (Revaluation state × Schedule) analyses on the effects of outcome revaluation were analyzed for each lesion group. Follow-up planned paired comparisons were Bonferroni corrected. An α = 0.05 was used for all analyses.

## Results

### Lesions of premotor cortex in mice

Ibotenic acid injections into M2 induced substantial damage within M2 (example shown in Figure 1A). Mice included in the study showed none or only slight lesion spread into surrounding cortices (primary motor cortex M1, and anterior cingulate Cg) (Figure 1A). To avoid any potential confound in the conclusions, mice with more extensive lesions to surrounding cortices (*n* = 7) were excluded from the behavioral analyses. Therefore, the final group sizes for the within-subject experiment were the following: *n* = 6 for Sham mice and *n* = 7 for M2 lesion mice. For mice trained solely on the RR schedule, group size was *n* = 7 for Sham mice, and *n* = 4 for M2 lesion mice: for mice trained exclusively on the RI schedule, group size was *n* = 7 for Sham mice and *n* = 6 for M2 lesion mice.

### Premotor cortex lesions do not affect acquisition of lever-press behavior

We first concurrently trained mice to lever-press a similar lever for the same food reward in two different contexts using different schedules that bias towards goal-directed (RR) versus habitual (RI) action strategies (Figure 1B) (see Methods, Gremel and Costa, 2013). M2 lesions had little effect on the acquisition of lever-press related behavior under either RI or RR schedules of reinforcement (Figure 1). A repeated-measures ANOVA (Lesion group × Training day) performed for each schedule showed both groups increased the number of lever presses (Figure 1C) (main effect of Training day; RI context: *F*_8,88_ = 9.78, *p* < 0.0001; RR context: *F*_8,88_ = 13.72, *p* < 0.0001) and response rate (Figure 1D) (main effect of Training day; RI context: *F*_8,88_ = 5.83, *p* < 0.0001; RR context: *F*_8,88_ = 6.36, *p* < 0.0001) across training under each schedule (no interactions or main effect Lesion group under either schedule). Further, Sham and M2 lesion mice earned similar rewards (Figure 1E) (no interaction or main effect of Lesion group, main effect of Training day; RI context: *F*_8,88_ = 10.39, *p* < 0.0001; RR context: *F*_8,88_ = 5.34, *p* < 0.0001) and made a similar number of head entries (Figure 1F) (no interaction or main effects) during RI and RR schedule training. These findings confirm previous reports (Yin, 2009), and suggest that lesions of mouse M2 cortex did not impair their ability to learn a new appetitive action, in this case a lever-press, to obtain a food reward.

### Premotor cortex is necessary for actions to be updated following outcome revaluation

Mice trained concurrently on RR and RI schedules underwent subsequent outcome revaluation testing to examine the sensitivity of lever-press behavior in each training context to changes in expected outcome value (Figure 1B). Planned ANOVAs (Revaluation state × Schedule) performed on lever-pressing for each lesion group showed that Sham mice selectively reduced the number of lever-presses only in the RR context following outcome devaluation, but had similar lever-presses between Valued and Devalued states in the RI context (Figure 1G) (interaction: *F*_1,22_ = 3.95, *p* = 0.05) (RR context: Bonferroni corrected *p* < 0.05; RI context *p* > 0.05). Hence, intact mice were able to shift between performing goal-directed actions in the RR context, and habitual actions in the RI context. In contrast, M2 lesion mice were habitual in both RI and RR training contexts, with no reduction in lever-presses in either context following outcome devaluation (Figure 1G) (interaction: *F*_1,24_ = 1.10, *p* > 0.3). M2 lesions did not affect consumption of either pellets or sucrose during outcome revaluation pre-feeding (no interaction; pellets Sham = 0.52 *g* ± 0.05, pellets M2 lesion = 0.60 *g* ± 0.18; sucrose Sham = 0.82 *g* ± 0.09, sucrose M2 lesion = 0.95 *g* ± 0.05) (*ps’* > 0.05). These data suggest that although lesioned mice were able to perform lever pressing, M2 lesions rendered the executed actions insensitive to changes in outcome value, and biased mice towards the use habitual action strategies.

### The effects of premotor cortex lesions cannot be attributed to deficits in using contextual information

**Figure 2:**
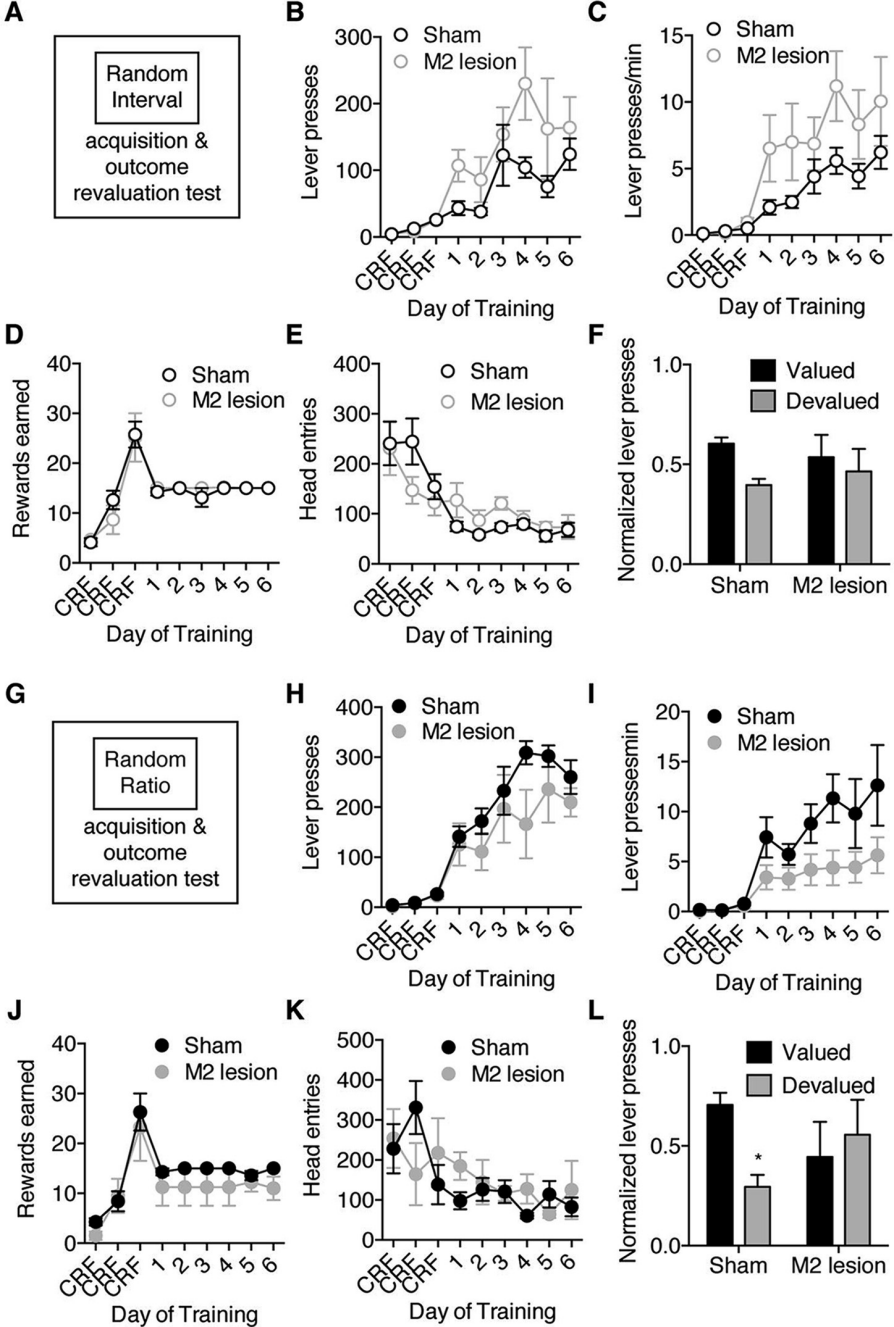
**M2 lesions disrupt goal-directed actions but spare habitual actions**. Separate groups of mice were trained to lever press for an outcome under only RI or only RR schedules of reinforcement, and then underwent subsequent outcome revaluation testing. **(A)** Schematic of experimental design. Mice were trained to press a lever only under a random interval (RI) reinforcement schedule, and then underwent outcome revaluation testing. **(B–F)** Effect of M2 lesions on acquisition under RI schedule on the average number of lever-presses made **(B)**, the average rate of lever-pressing **(C)**, the average rewards earned **(D)**, and the average head entries performed **(E)**. **(F)** Effect of outcome revaluation on normalized lever-pressing following RI schedule training for Sham and M2 lesion mice. **(G)** Schematic of experimental design. Mice were trained to press a lever only under a random ratio (RR) reinforcement schedule, and then underwent outcome revaluation testing** (H–K)** Effect of M2 lesions on acquisition under RR schedule on the average number of lever-presses made **(H)**, the average rate of lever-pressing **(I)**, the average rewards earned **(J)**, and the average head entries performed **(K)**. **(L)** Effect of outcome revaluation on normalized lever-pressing following RR schedule training for Sham and M2 lesion mice. For revaluation testing, Valued days = black bars, and Devalued day = grey bars. Error bars = ± SEM. * = Bonferroni corrected *p* < 0.05.

In the experiments described above the same animal learned to perform goal-directed pressing in one context and habitual pressing in another context. Therefore, one potential alternative explanation would be that M2 lesions interfered with the ability of mice to use contextual information to guide the shift between goal-directed and habitual action strategies. We therefore performed an experiment in which separate groups of Sham and M2 lesioned mice were trained on either RI or RR schedules of reinforcement (Figures 2A,G). Although M2 lesions appeared to interact differently with RR and RI lever-press acquisition, repeated-measures ANOVA (Lesion group × Training day) performed on acquisition data under each schedule did not reveal a significant effect of M2 lesions on the number of lever presses during acquisition in either the RI (Figure 2B) (main effect of Training day: *F*_8,96_ = 11.80, *p* < 0.0001; or RR (Figure 2H) (*F*_8,72_ = 27.40, *p* < 0.0001) schedules. Further, there were no significant effects of M2 lesions on response rate in either schedule (Figures 2C,I) (main effect of Training day; RI schedule only: *F*_8,96_ = 15.60,*p* < 0.0001; RR schedule only: *F*_8,72_ = 8.72, *p* < 0.0001). This was reflected in the lack of significant interactions between Lesion group and Training day, and unsupported by planned comparisons which did not reveal any significant differences. Sham and M2 lesion mice earned similar rewards across training under both schedules (Figures 2D,J) (main effect of Training day; RI schedule only: *F*_8,96_ = 24.90, *p* < 0.0001; RR schedule only: *F*_8,72_ = 21.04, *p* < 0.0001), and made similar head entries (Figures 2E,K) (main effect of Training day; RI schedule only: *F*_8,96_ = 11.62, *p* < 0.0001; RR schedule only: *F*_8,72_ = 3.20, *p* < 0.01) (no interactions or main effects of Lesion group). These data do show that M2 lesions did not grossly alter acquisition of lever-press behaviors when trained under only a RI or RR schedule. Still, a revaluation test showed M2 lesions did affect sensitivity to outcome devaluation in mice trained under a RR schedule of reinforcement (Figure 2L) (Repeated Measures ANOVA of Lesion group × Revaluation state, interaction: *F*_1,18_ = 5.96, *p* < 0.05). Sham mice trained to lever-press under a RR schedule reduced lever-presses in the devalued state (Bonferroni corrected *p* < 0.05), while M2 lesion mice made a similar number of lever-presses between valued and devalued states (*p* > 0.05). M2 lesions did not alter the sensitivity of actions trained under an RI schedule to changes in valued (Figure 2F) (interaction: *F*_1,22_ = 0.78, *p* > 0.05). Taken together, these results suggest that M2 is necessary for actions to be controlled by the expected outcome value.

## Discussion

By training the same mouse to shift between performing a similar action (lever pressing a similar lever for the same food outcome) using goal-directed versus habitual action strategies, we were able to investigate the contribution of M2 to the learning and performance of different action strategies in the same animal. M2 lesions did not affect the acquisition or performance of lever pressing *per se* in either schedule; no effects of M2 lesions were observed on the number of lever presses, response rate, number of earned outcomes, or head entry behavior. However, when we probed if animals learned (and used) the causal relationship between outcome obtainment and action by using outcome revaluation testing, we uncovered that M2 lesions prevented the use of the expected outcome value to control action execution. This suggests that M2 is necessary for goal-directed actions. We confirmed that the lesions effects were not caused by an inability of the animals to shift between contexts or schedules in an experiment where separated groups of mice were trained only on either RR or RI schedules of reinforcement. Once again, M2 lesions prevented the outcome revaluation induced- decrease in lever-pressing in RR trained mice, implicating M2 in goal-directed actions.

The lack of M2 lesion effects on actions trained under the RI schedule suggests that M2 processing is not necessary for the development and performance of habitual actions, which are more dependent on past reinforcement history, and do not reflect changes in expected outcome value (Dickinson, 1985; Balleine and Ostlund, 2007). The current findings provide further evidence (Gremel and Costa, 2013), that goal-directed and habitual actions are learned in parallel (not serially) and suggest that M2 is necessary for allowing the performed action to be controlled by its consequence. One should note that our M2 lesions do not extend throughout the entire M2 and it hence we cannot exclude the possibility that more complete lesions could alter habitual action strategies. Still, we did observe deficits in goal-directed actions suggesting the extent of the current lesions is sufficient to dissociate a role for M2 between goal-directed and habitual actions. It is not clear whether the present observation is due to an inability of mice to use goal-directed strategies for both the acquisition and expression of lever-press behavior. Our current results do suggest though, that M2 is necessary for the reflection of changing consequences in the execution of goal-directed actions.

Although rodent M2 is thought to be functionally akin to primate pre-SMA (Yin, 2009; Sul et al., 2011), relatively little has been done to directly investigate the role of this area in executive influence over motor planning in rodents compared to primates. The pre-SMA in primates has been implicated in task switching, response inhibition, and general motor learning and planning. In particular, task-switching is thought to involve pre-SMA functioning (Matsuzaka and Tanji, 1996; Shima et al., 1996; Dove et al., 2000; Rushworth et al., 2002; Isoda and Hikosaka, 2007). Indeed, findings in non-human primates have suggested that pre-SMA is involved in the switch from automatic to controlled behavior (Isoda and Hikosaka, 2007). The current findings suggest that a self-initiated action differentially recruits M2 depending on the causal structure guiding that action. While goal-directed actions seem to depend on M2, automatic or habitual action strategies do not seem to depend upon M2 function. Further, these findings suggest that unlike SMA (Padoa-Schioppa et al., 2002), at the level of M2 and pre-SMA executive processes beyond action kinematic processes contribute to motor planning. Recent work has also suggested a strong role for M2 in response inhibition (Obeso et al., 2013). In the present data, reduced goal-directed responding following outcome revaluation may involve such processes. Conversely, another possibility is that the lack of response inhibition observed following M2 lesions is due to a shift in action control, from goal-directed to habitual action strategies.

In light of the above discussion it is relevant to note that the present study examined self-paced actions. Previous studies using cued behaviors, for example a rewarded maze task cued by discriminative stimuli, found evidence of a role for M2 in value control over behavior selection (Sul et al., 2011). Using *in-vivo* recordings of neural activity in M2 as well as M2 lesions, the authors suggest that M2 is recruited during performance of value-controlled behavior. Although this study did not directly test this, the data could also be interpreted as showing a role for M2 in goal-directed behavior. Two previous studies has examined the role of M2 in isolated self-initiated actions examining sequence learning in rats (Ostlund et al., 2009) and in mice (Yin, 2009). The former using a two-action, two-outcome sequence task found a role for M2 in the use of sequences to guide goal-directed actions. M2 lesions disrupted the ability to use sequence-level action representations to guide goal-directed actions (Ostlund et al., 2009). Interestingly, M2 lesions did not disrupt the use of value control over actions when trained on two separate actions for two different outcomes (Ostlund et al., 2009). It could be that inhibiting a single action following devaluation recruits different neural mechanisms than choosing between the best of two outcomes (albeit one devalued) observed following training with two actions and two outcomes. Further, it may also be that the single-action, single-outcome design used in the present study is more sensitive to disruptions in the use of goal-directed action strategies.

In Yin (2009), M2 lesions severely impaired sequence learning and reversal learning, suggesting a role for mouse M2 in learning of serial order. Initial sequence learning may be a result of the learned positive relationship between response rate and reward rate thought to control acquisition of goal-directed actions (Dickinson and Balleine, 1994; Dezfouli and Balleine, 2012). That is, initial acquisition of the knowledge that response rate controls reward would be reflected in sequence formation. Further, it has been suggested that sequence formation is involved in the transition from initial goal-directed to habitual control over actions (Dezfouli and Balleine, 2012). In rodent at least, the dorsal lateral striatum is necessary for serial order learning and habit formation (Yin et al., 2004; Yin, 2010; Hilario et al., 2012; Gremel and Costa, 2013), suggesting a similar recruitment of neural circuits as routines become more crystallized. However, whether sequences themselves are necessary for acquisition and/or execution of goal-directed or habitual actions remains unknown. In addition, the present findings may also mirror the deficits previously observed in action sequence acquisition and reversal following M2 lesions (Yin, 2009). The impaired acquisition and reversal of serial order following M2 lesions in Yin (2009) may in part be explained by the loss of goal-directed action control over lever-pressing, and instead a reliance on habitual strategies which were not as effective for serial order learning. Further, while we did not examine the role of M2 specifically in action-outcome contingency learning, one could predict that M2 lesions would disrupt the ability to learn the new serial order following reversal. In relation to the current findings, if M2 lesion mice have impaired flexibility of learned lever-press behavior, it may in part explain the inability to reduce lever-pressing following changing consequences observed in the present data. Together, the present findings add to our knowledge of M2 involvement in executive control over self-initiated actions.

The present finding for a role of M2 in goal-directed actions adds to our knowledge of the underlying circuitry controlling self-initiated actions. Cortical and basal ganglia circuits have been identified in mediating goal-directed and habitual action strategies (Yin and Knowlton, 2006; Balleine and O’Doherty, 2009), with M2 joining additional lesion studies implicating the orbital frontal (OFC) (Gremel and Costa, 2013) and prelimbic cortices (Corbit, 2003) as well as mediodorsal nucleus (MD) of the thalamus (Corbit, 2003) and dorsal medial striatum in the control of goal-directed actions (Yin et al., 2005; Hilario et al., 2012; Gremel and Costa, 2013). In contrast, lesions to the infralimbic cortex (Killcross and Coutureau, 2003) and dorsal lateral striatum disrupt the use of habitual actions (Yin et al., 2004, 2006; Hilario et al., 2012; Gremel and Costa, 2013). M2 has been shown to directly project to dorsal striatum (Reep et al., 2003; Mitchell and Macklis, 2005; Pan et al., 2010) as well as to the OFC and MD (Reep et al., 1987; Hoover and Vertes, 2007). Also, it receives strong input from OFC, which may be important for updating action value (Reep et al., 1984; Hoover and Vertes, 2011; Gremel and Costa, 2013). Therefore, based on connectivity alone, one could hypothesize that interactions of M2 with these other brain regions of the circuitry would be involved in goal-directed actions.

In summary, the findings reported here present evidence for a role for mouse M2 in self-initiated goal-directed actions. While M2 lesions did not disrupt performance of the action, M2 lesions resulted in a bias towards habitual control over the action and disrupted the ability of actions to be controlled by their expected consequences. These results have important implications for understanding disease processes where actions are continually performed in spite of negative or unwanted consequences, such as obsessive-compulsive disorder and addiction-related behaviors.

## Conflict of interest statement

The authors declare that the research was conducted in the absence of any commercial or financial relationships that could be construed as a potential conflict of interest.
